# The Influence of NH_4_NO_3_ and NH_4_ClO_4_ on Porous Structure Development of Activated Carbons Produced from Furfuryl Alcohol

**DOI:** 10.3390/molecules27227860

**Published:** 2022-11-14

**Authors:** Agnieszka Kałamaga, Maria Carmen Román-Martínez, Maria Angeles Lillo-Ródenas, Rafał Jan Wróbel

**Affiliations:** 1 Department of Catalytic and Sorbent Materials Engineering, Faculty of Chemical Technology and Engineering, West Pomeranian University of Technology, Piastów 17 Ave., 70-310 Szczecin, Poland; 2 Department of Inorganic Chemistry, Materials Institute (IUMA), Faculty of Sciences, University of Alicante, Carretera de San Vincente del Raspeig s/n, 03690 Alicante, Spain

**Keywords:** activated carbons, carbonaceous materials, pore development, CO_2_ adsorption, CO_2_ uptake, supercapacitors, ethylene adsorption, furfuryl alcohol, hydrogen storage, methylene blue adsorption

## Abstract

The influence of NH_4_NO_3_ and NH_4_ClO_4_ on the porous texture and structure development of activated carbons produced from a non-porous polymeric precursor synthesized from furfuryl alcohol has been studied. The non-doped counterparts were prepared and studied for comparison purposes. NH_4_NO_3_ and NH_4_ClO_4_-doped polymers were carbonized under N_2_ atmosphere at 600 °C, followed by CO_2_ activation at 1000 °C and the obtained carbon materials and activated carbons were thoroughly characterized. The porosity characterization data have shown that NH_4_NO_3_-derived ACs present the highest specific surface area (up to 1523 m^2^/g in the experimental conditions studied), and the resulting porosity distributions are strongly dependent on the activation conditions. Thus, 1 h activation is optimum for the microporosity development, whereas larger activation times lead to micropores enlargement and conversion into mesopores. The type of doping salts used also has a substantial impact on the surface chemical composition, i.e., C=O groups. Moreover, NH_4_NO_3_ and NH_4_ClO_4_ constitute good sources of nitrogen. The type and contribution of nitrogen species are dependent on the preparation conditions. Quaternary nitrogen only appears in doped samples prepared by carbonization and pyrrolic, pyrydinic, and nitrogen oxide groups appear in the NH_4_NO_3_ -series. NH_4_NO_3_ incorporation has led to optimized materials towards CO_2_ and C_2_H_4_ sorption with just 1 h activation time.

## 1. Introduction

For several years, the main topic of researchers in activated carbons (ACs) has been developing porosity, which is one of the most important features of this type of material. The final porosity developed is often dependent on the used precursor. For the most part, activated carbons are produced from biomass like wood [1,2], stems [3], peels of fruits [4,5,6], or leaves [7]. Pores, which derive from the natural vascular system in this type of biomass precursors, are further developed in activated carbons obtained from them [8]. The second group of precursors is polymers, which are characterized by a non-porous structure and do not contain any impurities, such as silica or metal oxides, in their chemical composition [9,10]. Comparing these two kinds of precursors, it is claimed that biomass-derived activated carbons will have a more developed porous structure than polymer-derived carbonaceous materials because of the contained original pores in the former. Also, the less ordered structure of biomass precursors in comparison with polymeric ones explains that larger porosity usually developed in the former under comparable activation conditions [11]. However, in contrast to polymer precursors, biomass counterparts contain mineral matter, which could act as a catalyst and accelerate the decomposition of the precursor during pyrolysis [12,13]. Additionally, the ashes derived from mineral matter could block pores and interfere with the adsorption processes [14,15]. Moreover, the presence of ash in carbonaceous materials can constitute a disadvantage in their different applications, e.g., in the production of carbon electrodes. Therefore, the choice of an appropriate precursor, with pure chemical composition and highly developed porous structure, could pose an issue. One solution to this problem is using furfuryl alcohol as a precursor in ACs production. Furfuryl alcohol is produced from biomass via its dehydration followed by catalytic hydrogenation of furfural [16,17,18,19]. A highly pure chemical composition is the result of repeated distillation/extraction processes. To sum up, it constitutes a promising precursor to activated carbons with high porosity and pure chemical composition. Moreover, using this type of precursor ash removal processes prior to activation could be omitted. This allows for the reduction of production costs and consumption of chemical reagents (i.e., HCl or distilled water) [20,21].

Currently, methods of porous texture development are divided into two groups: physical and the so-called chemical activation [22,23,24,25,26,27,28]. Physical activation is usually conducted under CO_2_ or steam atmosphere at a temperature ranging from 800 °C to 1000 °C [29,30,31]. Rodriguez-Reinoso and co-workers reported that CO_2_ activation leads to narrow micropores creation and, subsequently, their widening occurs [32]. In the case of steam, the widening of pores occurs from the early stages of activation. Apart from the conventional method of heating, porosity could be developed through unconventional heating methods, such as microwave or plasma treatment. In comparison to conventional heating, microwave heating is conducted under milder conditions such as lower temperatures, shorter processing times, and requires less energy [33,34]. Additionally, this method implies fewer requirements for feedstock pre-treatment. The main difference between conventional and microwave heating lies in their mechanism. In the case of conventional heating, a particle is heated from the external surface to the core, whereas in microwave heating, a particle is heated from the core to the external surface. The second unconventional method of pores creation is plasma treatment. Plasma could be created from the following gases: oxygen, nitrogen, argon, ammonia, or air [35,36,37]. In this method, the surface of the sample is bombarded with ions or free radicals. It leads to the breaking of sp^2^ bonds in the carbon structure and the introduction of functional groups, which promote carbon etching. Inaccessible pores are opened and pore walls are collapsed. It causes an increase in porosity. Some advantages of plasma treatment include that it is faster than conventional heating and does not require solvents or toxic chemicals, being also highly efficient compared to chemical activation [35].

In the case of chemical activation methods, they are based on using mainly KOH, NaOH, K_2_CO_3_, H_3_PO_4_, or less frequently ZnCl_2_ [6,34,38,39,40,41,42,43,44,45,46,47,48,49,50,51,52,53]. Additionally, in carbonaceous materials preparation, some researchers used the following chemical compounds: FeCl_2_, NaCl, KCl, Fe_2_(SO_4_)_3_, (Na[Al(OH)_4_]), or NaSiO_3_ [54,55]. Recently, researchers are also paying attention to the influence of ammonium nitrate (NH_4_NO_3_) on porosity development. Subramanian and Viswanathan prepared activated carbon from sucrose and ammonium nitrate by carbonization in the temperature range from 600 °C to 900 °C [56]. They reported that activation with ammonium nitrate caused an increase in porosity. Apart from that, they checked the impact of the sucrose/ammonium nitrate mass ratio on pores development. Based on TGA-DTG analysis, they noted that the addition of ammonium nitrate increases the stability of the sample. The highest mass loss was observed at 438–448 °C. Bayrak et al. produced carbonaceous materials from rice husks for Cr^6+^, Cu^2+,^ and Ni^2+^ removal from water [57]. In their study, they used the following compounds as activating agents: NH_4_Cl, NH_4_Br, NH_4_I, (NH_4_)_2_HPO_4_, NH_4_HCO_3_ and NH_4_NO_3_. Comparing the mentioned compounds, ammonium nitrate caused the highest development of the porous structure. In contrast, the lowest impact on porosity was shown by ammonium iodide. Additionally, they noted that mesopores prevailed in activated carbons obtained by ammonium salts activation. Zheng and co-workers produced nitrogen/sulfur co-doped carbonaceous materials for supercapacitors application [7]. In their study, they utilized ginkgo leaves as precursors and examined the influence of ammonium nitrate on porous structure development. Additionally, they described the decomposition of ammonium nitrate, which takes place at temperatures over 220 °C. The authors claimed that activation is a redox reaction, and as a result of the increase of the heat-treatment temperature, ammonium nitrate is melted and partially decomposed to HNO_3(g)_, NH_3(g)_, N_2_O, and H_2_O_(g)_. Simultaneously, the oxidation reaction of biomass-derived carbon takes place. Gaseous products, such as CO_2_, H_2_O, NO_2_, and N_2_O, swell in graphene sheet-like carbon and further promote porous structure creation. In Table 1, specific surface areas of activated carbons obtained by ammonium nitrate activation are compiled.

As shown in the table above, all the activated carbons were prepared via chemical activation of biomass, in which original pores exist. Based on the literature, among the utilized chemical compounds containing NH_4_^+^, the ammonium nitrate ion has the highest impact on pores development. To the best of our knowledge, there are no scientific reports about ammonium perchlorate (NH_4_ClO_4_), which is also a strong oxidant. In industry, NH_4_ClO_4_ is commonly utilized as a solid rocket propellant. NH_4_ClO_4_ starts to decompose at 130 °C, and the main decomposition products include steam, nitrogen, oxygen, and chlorine [60,61]. From an environmental point of view, the produced chlorine leads to ozone layer degradation. For this reason, the produced chlorine should be adsorbed and utilized in other processes, such as in water disinfection tasks [62].

Taking all this into account, the general objectives of this study are to compare and examine the influence of ammonium nitrate and ammonium perchlorate on the preparation of activated carbon, paying attention to properties such as pore volume and distribution, specific surface area, size of crystallites and chemical composition. These carbonaceous materials will be prepared from furfuryl alcohol. Therefore, the impact of any silica and metal oxides, present in other precursors, on pore creation will be eliminated. The potential application of the obtained materials will be the adsorption of gases. Therefore, carbon dioxide (CO_2_) and ethylene (C_2_H_4_) will be used as model gases. Both gases negatively affect the environment. CO_2_ is one of the greenhouse gases whose emission is still increasing [63]. Currently, the concentration of CO_2_ in the atmosphere is 415 ppm. It mainly comes from anthropogenic sources, such as fossil fuel combustion or cement production. The high concentration of CO_2_ intensifies the greenhouse effect, which results in various climatic disasters, for example, droughts, melting of glaciers, and forest fires. C_2_H_4_ is known to be a plant hormone, which controls the ripening processes [64]. It is produced during the putrefactive processes of plants and leads to the faster ripening of vegetables that are nearby. For this reason, the regulation of the concentration of C_2_H_4_ is especially important in the case of vegetable and fruit storage in the food industry. Note that these selected molecules differ in their kinetic diameters. They are 0.33 nm and 0.39 nm for CO_2_ and C_2_H_4_, respectively.

## 2. Materials and Methods

### 2.1. Materials

In this study, the preparation scheme can be summarized as follows: either a mixture of furfuryl alcohol, maleic acid, and ethylene glycol (Mixture 1) or a mixture of furfuryl alcohol, maleic acid, and ethylene glycol with either NH_4_NO_3_ or NH_4_ClO_4_ (Mixture 2) led to polymeric materials, that were carbonized in a nitrogen atmosphere at 600 °C. The derived materials were characterized as obtained, and also after their activation with CO_2_ at 1000 °C for different holding times.

The samples obtained after carbonization were named AC in the Mixture 1 scheme, or either AC_NH_4_NO_3_ or AC_NH_4_ClO_4_ in the Mixture 2 carbonization scheme. Note that in the case of Mixture 2, this “carbonization” can really imply some chemical activation as well. Additionally, these materials were submitted to activation by CO_2_. In this case, their nomenclature includes a number representing the holding time of the CO_2_ activation step (in hours). A detailed description of the preparation scheme is shown next.

An activation precursor was prepared from 15 mL furfuryl alcohol (Nr. CAS: 98-00-0, Sigma Aldrich), 2 g maleic acid (Nr. CAS: 110-16-7, Chempur), and 14 mL ethylene glycol (Nr. CAS: 107-21-1, EUROCHEM). All reagents were mixed on the magnetic stirrer for 30 min at ambient temperature. Afterward, the mixture was heated in an oven from 40 °C to 200 °C. It was held for 10 min at 40 °C, 50 °C, 80 °C, and for 18 h at 200 °C. After heating, the obtained polymeric precursor was cooled at an ambient temperature and ground.

In the case of chemically activated materials, NH_4_NO_3_ (Nr. CAS: 6484-52-2, Chempur) or NH_4_ClO_4_ (prepared from ammonia water (Nr. CAS: 1336-21-6, STANLAB) and perchloric acid (Nr. CAS: 7601-90-3, Chempur)) salts were firstly dissolved in 14 mL ethylene glycol. An amount of 1 g NH_4_NO_3_ or 1 g NH_4_ClO_4_ was added. After that, the solutions were added to the furfuryl alcohol and maleic acid mixture (same quantities as in the previous conditions). All reagents were mixed on the magnetic stirrer for 30 min at an ambient temperature. Afterward, the mixture was heated in an oven from 40 °C to 200 °C. It was held for 10 min at 40 °C, 50 °C, 80 °C, and for 18h at 200 °C. After heating, each obtained precursor polymer was cooled at an ambient temperature and ground.

The three different precursors (either prepared with NH_4_NO_3_, NH_4_ClO_4,_ or without any of those) were subjected to a carbonization process under N_2_ atmosphere at 600 °C (temperature ramp 5 °C/min) for 4 h in an electric tube furnace (STF 15/180, Carbolite Gero). The flow of N_2_ was 100 mL/min. The three obtained materials were characterized as obtained and also submitted to a next step. In it, 1 g of each resulting sample was activated under CO_2_ atmosphere at 1000 °C (temperature ramp 5 °C/min) for 1 h, 2 h, or 3 h. The flow of CO_2_ was 50 mL/min during the whole process.

For simplicity, in some parts of the discussion these three sets of samples are referred to as “unmodified”, “NH_4_NO_3_ modified”, or “NH_4_ClO_4_ modified”.

The purity of N_2_ and CO_2_ were 5.0 and 4.5, respectively. Both gases were provided by Messer.

### 2.2. Methods

The porous structure of the obtained activated carbons was characterized using volumetric analysis (Autosorb, Quantachrome; Boynton Beach, USA). Pore volume and pore size distribution were determined based on N_2_ adsorption/desorption isotherms at −196 °C using the QSDFT model. Specific surface areas were calculated from the BET equation applied to the N_2_ adsorption data. Additionally, adsorption of CO_2_ at 0 °C allowed to determine the pores with diameters lower than 1.0 nm. In this case, the pore size distribution was determined using the NLDFT model. The porosity characterization data were calculated using QuadraWin software.

The structural properties of the ACs were analyzed using X-ray powder diffraction (Empyrean, PANalytical; Malvern, UK). Measurements were conducted in the 5°–80° 2θ range using copper radiation (K_α_ = 0.154056 nm). HighScore Plus software was used for XRD patterns interpretation and for stacking height (Lc) and lateral size (La) calculation.

Disorder degrees in the ACs were estimated using Raman spectroscopy analysis (Jasco NRS-5100; Easton, USA). Measurements were carried out with an Ar laser (wavelength = 532.11 nm, power = 2.0 mW, magnification of the objective = 20x). The activated carbons were scanned from 70 cm^−1^ to 3780 cm^−1^.

X-ray photoelectron spectroscopy was used to confirm the surface chemical composition of the carbons (PREVAC; Rogów, Poland). Functional groups were determined based on C 1s, O 1s, and N 1s signals. Data were calculated using CasaXPS 2.3.16 software.

Thermal stabilities of the carbonaceous materials were determined using a thermobalance (NETZSCH, STA 449F5; Selb, Germany). An amount of 10 mg of sample was heated from 50 °C to 950 °C with a temperature ramp of 20 °C/min. Measurements were conducted both under an argon or oxygen atmosphere. The flow of gases was 30 mL/min or 50 mL/min, respectively.

To test the ACs in their final application, adsorption measurements of CO_2_ and C_2_H_4_ were conducted on a homemade thermobalance operating under 1025 hPa. Firstly, 0.4 g of AC sample was heated to 250 °C to remove adsorbed compounds and moisture. The sample was held for 10 min at this temperature. After that, it was cooled to 30 °C under the investigated gas atmosphere. The flow of CO_2_ and C_2_H_4_ was 40 mL/min. Sorption capacities were calculated based on the mass difference between 250 °C and 30 °C.

## 3. Results and Discussion

### 3.1. Porous Structure

#### 3.1.1. N_2_ Adsorption at −196 °C

**Figure 1:**
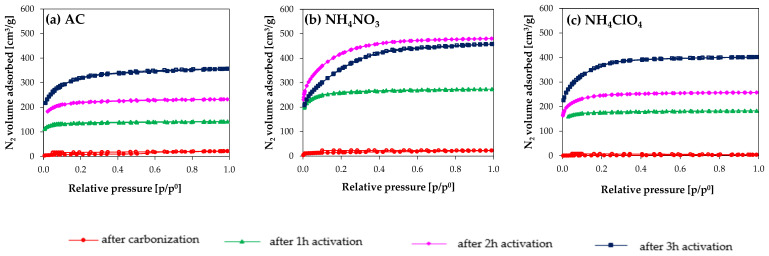
N_2_ adsorption/desorption isotherms at −196 °C of samples: (**a**) AC series, (**b**) NH_4_NO_3_ series, and (**c**) NH_4_ClO_4_ series.

N_2_ adsorption/desorption isotherms are presented in Figure 1. 

Figure 1 shows that the carbonization of the three different precursors leads to a very similar and poor porosity. Hence, the discussion is focused on data from samples after CO_2_ activation. According with the IUPAC classification, the obtained isotherms (Figure 1) are type I (b) [65]. They are characteristic of materials having wide micropores and narrow mesopores (<~2.5 nm). In this case, N_2_ adsorption is enhanced in the NH_4_ClO_4_ series and, especially, in the NH_4_NO_3_ series: comparing the influence of NH_4_NO_3_ and NH_4_ClO_4_ on N_2_ adsorption it can be seen that higher N_2_ adsorption was achieved in the NH_4_NO_3_-derived carbons.

**Figure 2:**
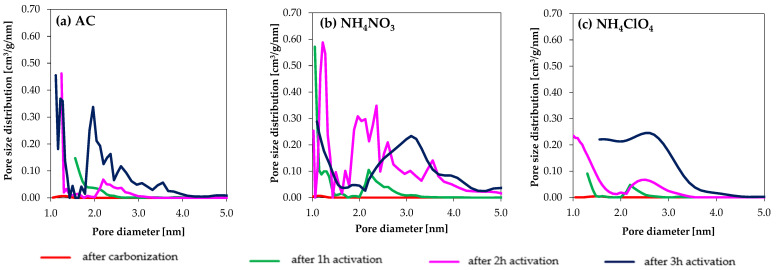
Pore size distribution of samples (**a**) AC series, (**b**) NH_4_NO_3_ series, (**c**) NH_4_ClO_4_ series calculated from N_2_ adsorption data at −196 °C.

Focusing on NH_4_NO_3_, larger N_2_ adsorption was observed in AC_NH_4_NO_3__2h in comparison to sample AC_NH_4_NO_3__3h, and also their porosity distributions were noticeably different. This was not observed in the AC and NH_4_ClO_4_-modified samples (carbonized in N_2_ and activated with CO_2_). The comparison between these two series highlights that the activation reaction in the NH_4_NO_3_ series proceeds in a much more efficient way than in the NH_4_ClO_4_ one. From a certain activation time (i.e., 3 h), a noticeable porosity widening is noticed in the NH_4_NO_3_ series, and even some porosity destruction, so the selection of the activation time is very important. The impact of activating salts on pore size distributions is presented in Figure 2.

This figure confirms that differences in the pore size distributions are noted for the three series of samples and, in general, the larger activation times lead to larger porosities.

In the ACs series, the highest porosity was reached in the sample AC_3h, whose mean pore size is around 2.0 nm. The mean pore size for NH_4_ClO_4__3h is around 2.5 nm, whereas it reaches 3.0 nm for NH_4_NO_3__3h.

Although higher pore volumes are in general present in the NH_4_NO_3_ series in comparison with the NH_4_ClO_4_ one, these differences minimize for 3 h activation time.

In Table 2, specific surface areas and pore volumes calculated from N_2_ adsorption data are presented.

This table shows that carbons obtained only through the “carbonization” process in N_2_ at 600 °C are characterized by low specific surface areas. In the case of AC, this sample is indeed obtained by carbonization. In AC_NH_4_NO_3_ and AC_NH_4_ClO_4_, some chemical activation may occur as well (due to the chemical compounds incorporated during the preparation of the precursor). This is especially remarkable in the AC_NH_4_NO_3_ sample, and explains the 57 m^2^/g of surface area reached.

The porosity values in AC_NH_4_NO_3_ and AC_NH_4_ClO_4_ series are lower than, for example, those reported by Bayrak and co-workers, who used rice husks as precursors [57]. Although the activation temperature is the same in both cases, the higher specific surface areas reported by Bayrak et al. may be very much influenced by the different nature of the precursor.

Sample AC_NH_4_NO_3__2h reached the highest BET specific surface area (1523 m^2^/g) and the highest volume of micropores (0.41 cm^3^/g). After the extension of the activation time to 3 h, the volume of micropores decreased by half and the mesopore volume increased. In the case of the NH_4_ClO_4_ series, the highest specific surface area and pores volume was exhibited by sample AC_NH_4_ClO_4__3h (1342 m^2^/g and 0.29 cm^3^/g, respectively). Comparing the effect of NH_4_NO_3_ and NH_4_ClO_4_, it has been found that NH_4_NO_3_ is more efficient for porosity development.

It is difficult to compare the ACs produced in the present study with those from other papers. For example, Mu et al. produced activated carbons by hydrothermal method followed by KOH activation at 800 °C for 1 h [59]. The highest specific surface area reached was 1976 m^2^/g. It must be noted that so-developed porous structure is partially related to using KOH, one of the most effective activating agents, but also the proportion of chemical agent needs to be taken into consideration.

#### 3.1.2. CO_2_ Adsorption at 0 °C

**Figure 3:**
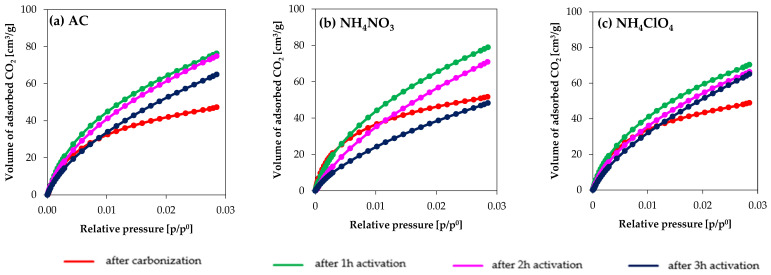
CO_2_ adsorption/desorption isotherms at 0 °C for (**a**) AC series, (**b**) NH_4_NO_3_ series, (**c**) NH_4_ClO_4_ series.

Figure 3 presents the CO_2_ adsorption isotherms at 0 °C for the different samples, which shows, that, in general, CO_2_ adsorption is lowest for the carbonized samples, whereas those activated for 1 h activation show the highest CO_2_ adsorption. Increasing the activation time beyond 1 h leads to a decrease in the narrow micropores (characterized by CO_2_ adsorption), being such a decrease is especially remarkable for NH_4_NO_3__3h. The similarity in the AC and NH_4_ClO_4_ series remarks that no benefit, from the point of view of narrow micropore development, occurs by incorporating this chemical agent into the composition of the precursor, and only NH_4_NO_3__1h is interesting from that point of view.

**Figure 4:**
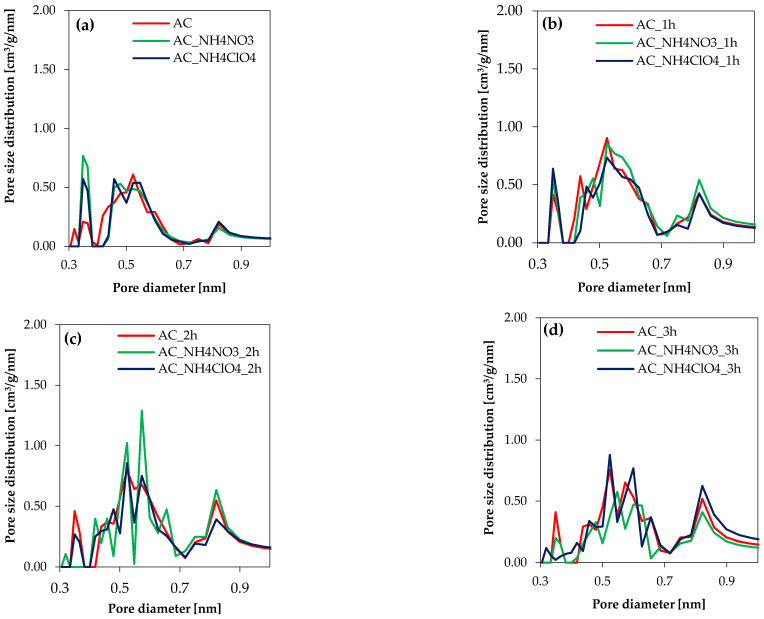
Micropore size distributions based on CO_2_ adsorption data at 0 °C for (**a**) just-carbonized carbons, (**b**) activated carbons after 1 h activation, (**c**) activated carbons after 2 h activation, (**d**) activated carbons after 3 h activation.

Figure 4 highlights that from the point of view of the CO_2_ pore size distributions, there are no important differences between the series. More remarkably, in the just-carbonized carbons, there is an increase in the volume of pores in the diameter range from 0.3 nm to 0.7 for AC_NH_4_NO_3_ and AC_NH_4_ClO_4_. After activation, micropore size distributions were comparable for all the obtained samples and the differences between them were negligible.

Micropore specific surface areas and micropores volumes determined by CO_2_ adsorption at 0 °C are presented in Table 3.

Table 3 confirms that the addition of oxidizing salts to the composition of the precursors caused insignificant changes in the narrow microporous structure: comparing the AC, NH_4_NO_3,_ and NH_4_ClO_4_ series, noticeable differences were not observed. The highest microporous specific surface area and micropores volume were exhibited by the sample AC_NH_4_NO_3__1h. They were 724 m^2^/g and 0.29 cm^3^/g, respectively.

### 3.2. Structural Properties

#### 3.2.1. X-ray Diffraction Analysis

**Figure 5:**
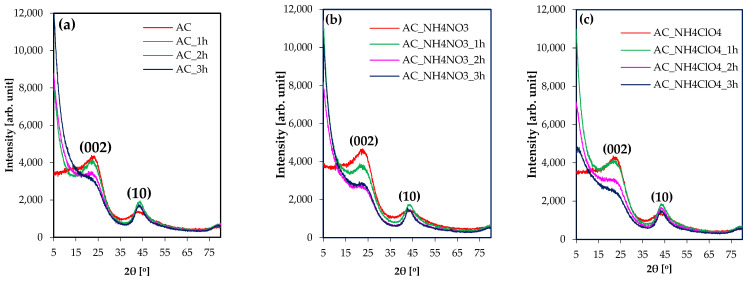
XRD-patterns of (**a**) AC series, (**b**) NH_4_NO_3_ series, (**c**) NH_4_ClO_4_ series.

XRD patterns for all obtained activated carbons are presented in Figure 5. They present two main reflections at positions 24° and 45°. Their Miller indexes are (002) and (10), respectively [66].

In the case of just-carbonized carbons, peaks for (002) reflection, which corresponds to the spacing between graphenic planes, are high and wide. Activation processes lead to their intensity decreasing and peak narrowing with the extension of the activation time. A more pronounced (002) reflection decrease (i.e., for 3 h activation time with respect to the carbonized sample) is observed in the NH_4_NO_3_ series in comparison to AC and NH_4_ClO_4_ ones.

For all the obtained just-carbonized samples, (10) reflections are broad and smooth. The position of (10) reflections is changing with the lateral size of carbon pieces. This feature, and the origin of (10) reflection are very well described in detail elsewhere [66,67].

1 h activation processes led to the increase of the intensity of peaks corresponding to those reflections, becoming narrower and sharper. Moreover, the extension of the activation time caused a slight decrease in the intensity of the peaks associated to the (10) reflections.

Based on obtained XRD patterns, stacking height (Lc) and lateral size (La) of carbon crystallites were calculated from the following equations:

(1) $$\mathrm{Lc}=\frac{0.89\lambda }{B_{c}cos\theta_{c}}$$

(2) $$\mathrm{La}=\frac{1.89\lambda }{B_{a}cos\theta_{a}}$$

where *θ* is the angle of a reflection, *λ* is wavelength of X-rays, and *Bc* and *Ba* are full widths at half maximum (FWHM) of (002) and (10) reflections, respectively. 

Stacking height (Lc), lateral size (La) and interlayer distance (d_002_) of carbon crystallites are presented in Table 4. 

These data show that modification of the parent polymers with the NH_4_NO_3_ or NH_4_ClO_4_ salts caused substantial changes in the stacking height of carbon crystallites during the carbonization process, whereas the lateral size was not affected. In comparison with AC, AC_NH_4_ClO_4_ shows a decrease in stacking height from 1.0 nm to 0.7 nm. In the case of AC_NH_4_NO_3_, this difference was only 0.1 nm.

After the activation processes, the lateral size significantly increased, whereas the stacking height increased only slightly. Sample AC_NH_4_NO_3__3h exhibited the highest value of Lc and La. They were 1.2 nm and 3.5 nm, respectively. The same material presented the highest volume of mesopores (0.39 cm^3^/g). In the case of sample AC_NH_4_NO_3__2h, which has the highest specific surface area, Lc and La values are 1.2 nm and 3.3 nm, respectively. Therefore, an increase of lateral size could also be related to the development of porosity.

#### 3.2.2. Raman Spectroscopy

The ordered and defective carbon structures in the carbonaceous materials were estimated on the basis of the intensity ratio of D-band to G-band (I_D_/I_G_). The formation of the D-band appears near 1345 cm^−1^ and is related to disordered structure. On the other hand, the G-band is associated with the regular structure of carbon and appears near 1596 cm^−1^. The crystallites size (La) [68,69,70] were determined based on the following equation, and are compiled in Table 5:

(3) $$\mathrm{La}=\frac{C}{R}$$

where *R* is the I_D_/I_G_ intensity ratio of D-band to G-band and constant *C* is 35 in this study.

The carbonized samples prepared with the doped polymers show a higher disordered structure than the one prepared with the undoped polymer. Comparing the influence of the type of salts, it can be noted that carbons from the NH_4_NO_3_ series are characterized by a more disordered structure than those from NH_4_ClO_4_ series. After the activation processes, the disordered structure significantly increased. The highest increase in I_D_/I_G_ ratio was exhibited after 3 h activation by all the obtained samples. The I_D_/I_G_ ratio for them was 1.09.

The largest size of crystallites (La) was exhibited by the sample AC. It was 5.9 nm. Significantly lower La values were exhibited by the carbonized samples from NH_4_NO_3_ and NH_4_ClO_4_ series. They were 4.9 nm and 5.1 nm, respectively. Further activation processes caused a decrease in the size of carbon crystallites. Moreover, this results in similar sizes of crystallites. The lowest carbon crystallites were exhibited by all the samples after 3 h activation.

The structural parameters La obtained by Raman are in good agreement with La determined by the XRD method.

### 3.3. Surface Chemical Composition

Table 6 summarizes the surface composition of the prepared ACs, determined by XPS analysis. Hydrogen is also present in the chemical composition of the ACs, but it cannot be detected by XPS.

Based on the surface chemical composition analysis by XPS, it can be noted that the preparation of ACs with this procedure, which includes the precursor ‘doping’ with NH_4_NO_3_ or NH_4_ClO_4_, is suitable for the incorporation of nitrogen heteroatoms in the composition of the ACs. Thus, the corresponding NH_4_NO_3_ and NH_4_ClO_4_-ACs contain 2–4 at. % of nitrogen on their surface and the concentrations of nitrogen are similar for both NH_4_NO_3_ and NH_4_ClO_4_ series.

These ACs also possess noticeable surface oxygen contents. The highest concentration of oxygen was exhibited by the sample named AC. Comparing the just-carbonized NH_4_NO_3_ and NH_4_ClO_4_ series, it can be observed that sample AC_NH_4_NO_3_ has a higher concentration of oxygen than AC_NH_4_ClO_4_. Further activation of the NH_4_NO_3_ series leads to lower oxygen concentration than in the case of the NH_4_ClO_4_ series. Chlorine is not detected in the AC_NH_4_ClO_4_ obtained materials. It is assumed that it was removed as a gaseous byproduct during the carbonization process.

**Figure 6:**
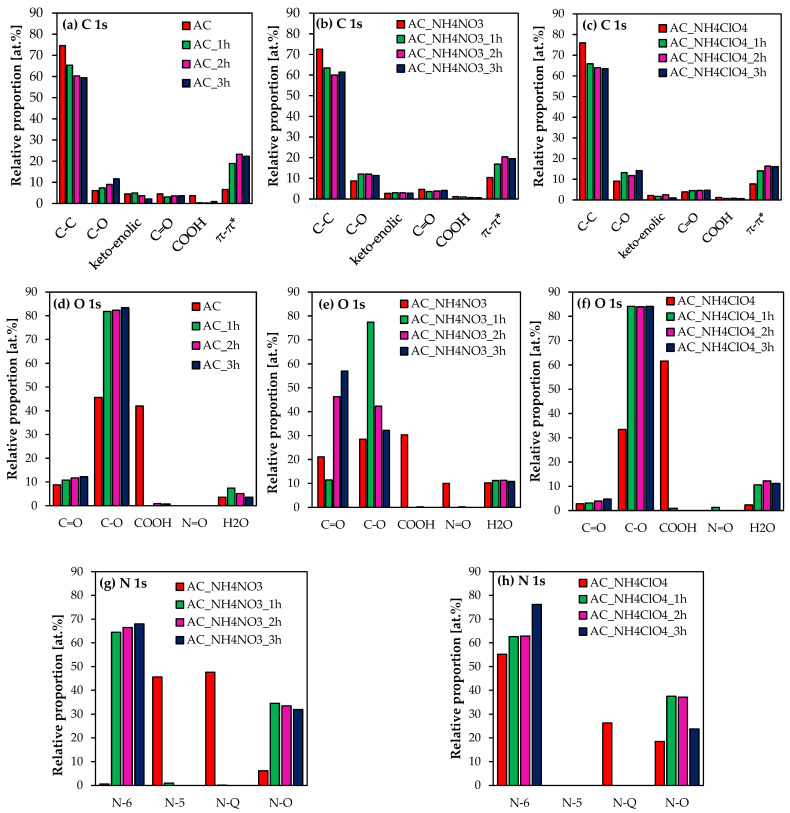
Carbon functional groups (**a**–**c**), oxygen functional groups (**d**–**f**), nitrogen functional groups (**g**,**h**) on the surface of the obtained activated carbons.

The XPS signals of carbon, oxygen, and nitrogen have allowed determining the type and the concentration of functional groups appearing on the surface of the obtained activated carbons. They are presented in Figure 6.

Functional groups containing carbon were identified based on the following binding energies: C-C 284.6 ± 0.3 eV, C-O 286.1 ± 0.3 eV, keto-enolic 286.4 ± 0.3 eV, C=O 287.6 ± 0.3 eV, COOH 289.1 ± 0.3 eV and π-π* (satellite peak of carbon in aromatic compounds, C-C with sp^2^ hybridization) 292.15 ± 2.15 eV. The full width at half maximum (FWHM) was set at the same value for each functional group, except for the satellite peak. In the case of O 1s signal, binding energies for particular components were: C=O 531.1 ± 0.3 eV, C-O 532.8 eV± 0.3 eV, COOH 534.2 ± 0.3 eV, N=O 535.2 ± 0.3 eV, and gas phase H_2_O 536.1 ± 0.3 eV. Nitrogen functional groups were determined based on the following binding energies: pyrydinic N-6 397.8 ± 0.3 eV, pyrrolic N-5 399.9 ± 0.3 eV, quaternary N-Q 401.2 ± 0.3 eV, and nitrogen oxides N-O 402.2 ± 0.3 eV. In the case of O 1s and N 1s, full width at maximum (FWHM) was set at the same value for each component.

Based on the deconvolution of C 1s, O 1s, and N 1s signals, it can be observed that the obtained AC series contain mainly C-C (sp^3^ and sp^2^ hybridization) and C-O bonds.

The exception is sample AC, which also contains COOH groups. However, after activation processes, COOH groups decomposed into C=O and C-O bonds. It is assumed that the high concentration of COOH groups is associated with maleic acid, used as a catalyst during polycondensation reactions to prepare the precursor.

In the case of carbons derived from doped-precursors, the addition of NH_4_NO_3_ caused a substantial increase in the content of C=O groups and a decrease of C-O groups. In the case of the NH_4_ClO_4_ series, it led to lower concentrations of C=O groups in comparison to the AC series and NH_4_NO_3_ series. On the other hand, the NH_4_ClO_4_-activated carbons are characterized by higher C-O groups concentration than the NH_4_NO_3_-activated carbons.

In the case of nitrogen functional groups, NH_4_NO_3_- and NH_4_ClO_4_-modified activated carbons contain mainly pyrydinic (N-6) and nitrogen oxide (N-O) groups. Quaternary nitrogen (N-Q) only appears in samples prepared by carbonization.

Moreover, NH_4_NO_3_-activated carbons contain pyrrolic nitrogen (N-5), which is not present in NH_4_ClO_4_-activated carbons. Focusing on the quaternary nitrogen concentration, in the NH_4_NO_3_ series it is higher than in the NH_4_ClO_4_ series.

### 3.4. Thermal Stability

**Figure 7:**
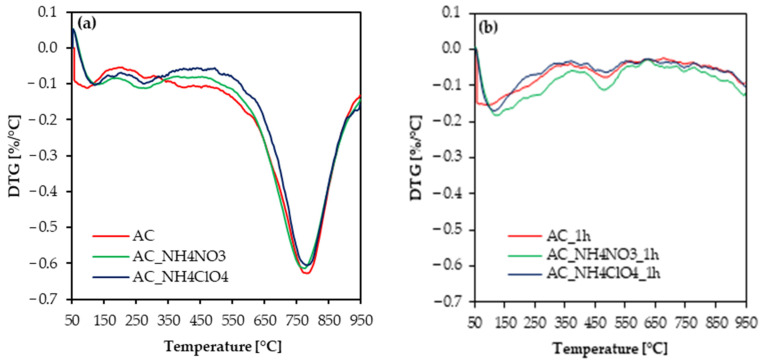
DTG data for determining thermal stability for (**a**) carbons only after carbonization, (**b**) carbons after 1 h activation.

Figure 7 compiles the DTG data for either samples obtained after carbonization or after carbonization and activation for 1 h.

In the samples carbonized at 600 °C two peaks, attributed to the removal of volatile organic compounds and adsorbed moisture, appear at low temperatures. The third peak, appearing between 550 °C and 780 °C, helps to understand the stability temperature of the carbonized activated carbons. The DTG data shows that after 1 h activation process, activated carbons have comparable thermal stabilities. Thus, the addition of the NH_4_NO_3_ or NH_4_ClO_4_ during the synthesis of the precursor does not modify the thermal stability of the obtained samples.

It is worth highlighting that these loss peaks correspond to the COOH groups removal, which is confirmed by the XPS analysis.

### 3.5. CO_2_ and C_2_H_4_ Adsorption

**Figure 8:**
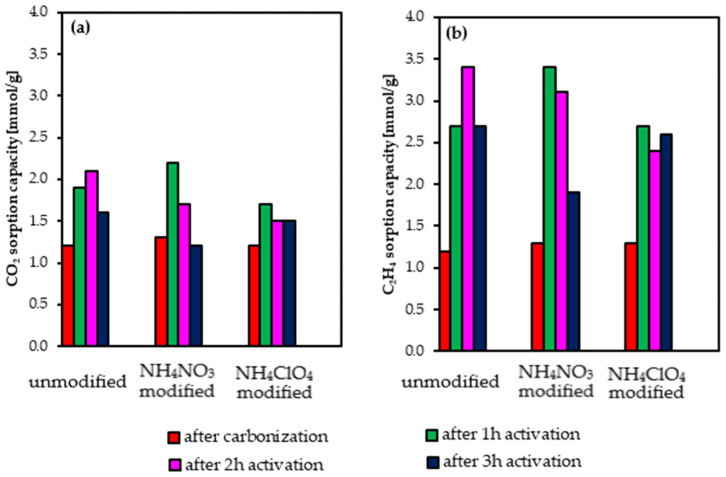
CO_2_ (**a**) and (**b**) C_2_H_4_ uptakes at 30 °C.

CO_2_ and C_2_H_4_ sorption capacities were measured at a temperature of 30 °C. In Figure 8, these capacities are presented. 

Figure 8 shows that for all the activated carbons, the C_2_H_4_ sorption capacity is substantially higher than of CO_2_. The carbonized materials show similar and lower CO_2_ and C_2_H_4_ uptakes (~1.3 mmol/g). The highest adsorption of CO_2_ was exhibited by the sample AC_NH_4_NO_3__1h (2.2 mmol/g). In the unmodified AC series, the highest sorption capacity of CO_2_ was exhibited by the sample AC_2h (2.1 mmol/g). So, the incorporation of NH_4_NO_3_ to the precursor has led to an increase in the sorption properties. The highest C_2_H_4_ adsorption was reached by the samples AC_2h and AC_NH_4_NO_3__1h. It was 3.4 mmol/g.

These results highlight that the incorporation of NH_4_NO_3_ into the composition of the precursor permits to shorten the activation time: it leads to optimized materials towards CO_2_ and C_2_H_4_ sorption with just 1 h activation time (comparable CO_2_ and C_2_H_4_ sorption capacities are attained from non-doped precursor-activated carbon after 2 h activation).

Figure 8a,b show that the increase in the activation time beyond 2 h for the AC series and beyond 1 h for the NH_4_NO_3_ and NH_4_ClO_4_ series leads to a decrease in their sorption capacities of CO_2_ and C_2_H_4_. Comparison of these results with the characterization data of the different activated carbons highlight that porosity, particularly narrow microporosity, seems to be the property determining the CO_2_ and C_2_H_4_ sorption capacities.

Note that the CO_2_ and C_2_H_4_ sorption capacities for the prepared ACs are similar to those from the literature for biomass-derived activated carbons. For instance, Zhang and co-workers produced carbon adsorbents from soybean straw via pyrolysis at 500 °C followed by CO_2_ activation at 900 °C [71]. As a result, CO_2_ uptake at 30 °C reached 1.5 mmol/g.

Xiong et al. prepared activated carbons from cotton stalks by CO_2_ activation at 900 °C [72]. The achieved sorption capacity of CO_2_ at 20 °C was 1.8 mmol/g.

On the other hand, Lahijani proposed the preparation of activated carbons from walnut shells via pyrolysis at 900 °C and modification with magnesium [73]. In their case, CO_2_ uptake at 25 °C reached 1.9 mmol/g.

Wang et al. obtained carbonaceous materials for the C_2_H_4_ adsorption using hardwood ligninosulfate as a precursor [74]. The highest C_2_H_4_ uptake was reached by the sample obtained after pyrolysis at 800 °C. It was 2.2 mmol/g at 25 °C. Ye and co-workers measured the sorption capacities of C_2_H_4_ and propylene (C_3_H_6_) for 15 commercial activated carbons [75]. In the case of C_2_H_4_, the highest uptake was 3.1 mmol/g.

## 4. Conclusions

The purpose of this study was to examine the influence of NH_4_NO_3_ and NH_4_ClO_4_ added during the preparation of a furfuryl-derived precursor on the preparation of ACs by “carbonization” followed by CO_2_ activation. It has been shown that the addition of NH_4_NO_3_ to the precursor’s composition has led to an important porosity enhancement, with specific surface areas up to 1523 m^2^/g, micropore volumes up to 0.41 cm^3^/g, mesopore volumes up to 0.29 cm^3^/g (and some slight increase in narrow microporosity for a low activation time).

The incorporation of NH_4_NO_3_ and NH_4_ClO_4_ does not influence the thermal stability of the prepared carbons but modifies the composition of the surface groups. Thus, an advantage of these modified ACs is that they present surface nitrogen contents between 2–4 at. % and they maintain high surface oxygen contents. The type and contribution of nitrogen species are dependent on the preparation conditions. Quaternary nitrogen only appears in doped samples prepared by carbonization, being larger for NH_4_NO_3_. NH_4_NO_3_-activated carbons contain pyrrolic nitrogen, not present in NH_4_ClO_4_-activated carbon, and both NH_4_NO_3_- and NH_4_ClO_4_-modified activated carbons contain pyrydinic and nitrogen oxide groups.

The prepared ACs present a good structural order, derived from the corresponding carbonized materials. Comparing the influence of the doping salts, carbons in NH_4_NO_3_ series are characterized by a more disordered structure than from the NH_4_ClO_4_ series, presenting both series of samples higher disordered structure than the one prepared from the undoped polymer.

The highest adsorption of CO_2_ was exhibited by the sample AC_NH_4_NO_3__1h (2.2 mmol/g). In the unmodified AC series, the highest sorption capacity of CO_2_ was exhibited by the sample AC_2h (2.1 mmol/g). The highest C_2_H_4_ adsorption was reached by the samples AC_2h and AC_NH_4_NO_3__1h. It was 3.4 mmol/g. These two ACs present the largest amount of micropores with a mean diameter of around 0.5–0.7 nm, particularly interesting for these applications.

NH_4_NO_3_ is commonly used as a fertilizer and its cost is relatively low. The precursor’s modification with NH_4_NO_3_ reduces the activation time from 2 h to 1 h, with the consequent reduction of the production costs, leading to activated carbons with comparable sorption capacities of CO_2_ and C_2_H_4_ to carbons derived from undoped precursors.

## Figures and Tables

**Table 1:** Comparison of the specific surface area of activated carbons obtained through ammonium nitrate activation.

Sample	Preparation Parameters	Specific Surface Area [m^2^/g]	Ref.
C_NA900_	Sucrose/NH_4_NO_3_ mass ratio 3:1 (C_NA900_) and 2:1 (C_NB900_). Carbonization under N_2_ atmosphere for 6 h at 900 °C.	489	[56]
C_NB900_		518	
AC_NH_4_NO_3_	Rice husk/NH_4_NO_3_ mass ratio: 1:4 Carbonization under N_2_ atmosphere for 0.5h at 600 °C.	457	[57]
AN/GL_700_	Ginkgo leaves/NH_4_NO_3_ mass ratio: 1:2. Carbonization under N_2_ atmosphere for 1 h at 700 °C.	672	[7]
PWAC	Pistachio wood wastes/NH_4_NO_3_ impregnation ratio: 5% wt. Pyrolysis under N_2_ atmosphere at 800 °C for 2 h.	1448	[58]
N-carbon_700_	Cellulose/NH_4_NO_3_/distilled water mass ratio 1:1:10. Hydrothermal carbonization at 240 °C for 5 h. Activation with KOH at 700 °C and 800 °C for 1 h.	1184	[59]
N-carbon_800_		1976	

**Table 2:** Specific surface areas and pores volume in the obtained activated carbons.

Sample	SSA ^1^ [m^2^/g]	V_total_ [cm^3^/g]	V_micro_ ^2^ [cm^3^/g]	V_meso_ ^3^ [cm^3^/g]
AC	28	0.03	0.00	0.03
AC_1h	535	0.22	0.19	0.01
AC_2h	843	0.36	0.30	0.03
AC_3h	1174	0.55	0.36	0.15
AC_NH_4_NO_3_	57	0.04	0.02	0.02
AC_NH_4_NO_3__1h	999	0.43	0.34	0.04
AC_NH_4_NO_3__2h	1523	0.75	0.41	0.28
AC_NH_4_NO_3__3h	1292	0.71	0.26	0.39
AC_NH_4_ClO_4_	6	0.01	0.00	0.00
AC_NH_4_ClO_4__1h	696	0.28	0.24	0.02
AC_NH_4_ClO_4__2h	933	0.40	0.27	0.09
AC_NH_4_ClO_4__3h	1342	0.62	0.29	0.26

^1^ SSA—The specific surface area, calculated by the BET equation. ^2^ V_micro_—The volume of pores with diameter lower than 2 nm, determined from N_2_ adsorption. ^3^ V_meso_—The volume of pores with diameter in the range from 2 nm to 50 nm, estimated from N_2_ adsorption.

**Table 3:** CO_2_ micropore specific surface areas and micropore volumes for the prepared ACs.

Sample	CO_2_ Micropore Specific Surface Area ^1^ [m^2^/g]	V_total_ [cm^3^/g]	V_0_._7 nm_ ^2^ [cm^3^/g]	V_0_._8 nm_ ^3^ [cm^3^/g]	V_1_._0 nm_ ^4^ [cm^3^/g]
AC	454	0.15	0.10	0.10	0.12
AC_1h	706	0.27	0.15	0.16	0.21
AC_2h	686	0.27	0.14	0.16	0.21
AC_3h	589	0.25	0.12	0.14	0.19
AC_NH_4_NO_3_	496	0.16	0.11	0.11	0.13
AC_NH_4_NO_3__1h	724	0.29	0.15	0.17	0.22
AC_NH_4_NO_3__2h	640	0.27	0.13	0.15	0.21
AC_NH_4_NO_3__3h	435	0.19	0.09	0.10	0.14
AC_NH_4_ClO_4_	471	0.15	0.10	0.10	0.13
AC_NH_4_ClO_4__1h	651	0.25	0.14	0.15	0.19
AC_NH_4_ClO_4__2h	600	0.25	0.13	0.14	0.19
AC_NH_4_ClO_4__3h	587	0.27	0.11	0.13	0.20

^1^ SSA_CO2_—The micropore specific surface area obtained on the basis on CO_2_ adsorption at 0 °C. ^2^ V_0_._7 nm_—The volume of pores with diameter up to 0.7 nm., determined from CO_2_ adsorption. ^3^ V_0_._8 nm_—The volume of pores with diameter up to 0.8 nm., determined from CO_2_ adsorption. ^4^ V_1_._0 nm_—The volume of pores with diameter up to 1.0 nm., determined from CO_2_ adsorption.

**Table 4:** Stacking height (Lc), lateral size (La) and interlayer distance (d_002_) of carbon crystallites.

Sample	Lc [nm]	La [nm]	d_002_ [nm]
AC	1.0	1.9	0.381
AC_1h	0.9	3.1	0.392
AC_2h	1.1	3.2	0.387
AC_3h	0.9	3.2	0.388
AC_NH_4_NO_3_	0.9	1.9	0.394
AC_NH_4_NO_3__1h	1.1	3.1	0.381
AC_ NH_4_NO_3__2h	1.2	3.3	0.373
AC_ NH_4_NO_3__3h	1.2	3.5	0.371
AC_NH_4_ClO_4_	0.7	1.9	0.417
AC_ NH_4_ClO_4__1h	1.0	3.1	0.388
AC_ NH_4_ClO_4__2h	1.0	3.3	0.387
AC_ NH_4_ClO_4__3h	1.1	3.3	0.384

**Table 5:** I_D_/I_G_ ratios and carbon crystallites (La) size of obtained activated carbons.

Sample	I_D_/I_G_ Ratio	D-Band Width [cm^−1^]	G-Band Width [cm^−1^]	La [nm]
AC	0.60	272.81	77.48	5.9
AC_NH_4_NO_3_	0.71	283.99	85.86	4.9
AC_NH_4_ClO_4_	0.68	256.92	96.28	5.1
AC_1h	1.09	194.63	93.65	3.2
AC_NH_4_NO_3__1h	0.93	220.33	94.07	3.8
AC_NH_4_ClO_4__1h	0.97	212.37	94.17	3.6
AC_2h	1.03	169.21	81.10	3.4
AC_NH_4_NO_3__2h	1.01	202.89	91.47	3.5
AC_NH_4_ClO_4__2h	0.95	201.71	90.14	3.7
AC_3h	1.09	179.18	83.17	3.2
AC_NH_4_NO_3__3h	1.09	191.59	87.11	3.2
AC_NH_4_ClO_4__3h	1.09	193.59	89.99	3.2

**Table 6:** Surface chemical composition, determined using XPS, for the obtained activated carbons.

Sample	Concentration [at. %]		
	C	O	N
AC	87	13	0
AC_1h	97	3	0
AC_2h	97	3	0
AC_3h	96	4	0
AC_NH_4_NO_3_	88	9	3
AC_NH_4_NO_3__1h	91	6	3
AC_NH_4_NO_3__2h	89	8	3
AC_NH_4_NO_3__3h	87	9	4
AC_NH_4_ClO_4_	90	7	3
AC_NH_4_ClO_4__1h	91	8	2
AC_NH_4_ClO_4__2h	90	8	2
AC_NH_4_ClO_4__3h	85	13	3

## Data Availability

The data presented in this article will be available upon request.

## Sample Availability

Samples of the compounds are available in limited amount from the author.
